# Gamma irradiation of adenine and guanine adsorbed into hectorite and attapulgite

**DOI:** 10.1016/j.heliyon.2023.e16071

**Published:** 2023-05-06

**Authors:** A. Meléndez-López, J. Cruz-Castañeda, A. Negrón-Mendoza, S. Ramos-Bernal, A. Heredia, L.G. Castro-Sanpedro, D. Aguilar-Flores

**Affiliations:** a Instituto de Ciencias Nucleares, Circuito Exterior s/n, Ciudad Universitaria, Col. Universidad Nacional Autónoma de México, Deleg. Coyoacán, Apartado Postal 70-543, C.P. 04510, CDMX, Mexico; b Facultad de Estudios Superiores Zaragoza Campus 2. Batalla 5 de Mayo s/n, Ejército de Oriente Zona Peñón, Iztapalapa, 09230, CDMX, Mexico

**Keywords:** Nitrogenous bases, Clays, Adsorption–desorption processes, Gamma irradiation, Chemical evolution

## Abstract

This study focuses on the radiolysis (up to 36 kGy) of guanine and adenine (nitrogenous bases) adsorbed in hectorite and attapulgite to highlight the potential role of clays as protective agents against ionizing radiation in prebiotic processes. In this framework, the study investigated the nitrogenous bases’ behavior in two types of systems: a) aqueous suspension of adenine–clay systems and b) guanine–clay systems in the solid state. This research utilized spectroscopic and chromatographic techniques for its analytical purposes. Regardless of the reaction medium conditions, the results reveal that nitrogenous bases are stable under ionizing irradiation when adsorbed on both clays.

## 1 Introduction

John D. Bernal and Victor M. Goldschmidt independently postulated that clays have a role in chemical evolution [1,2]. Many studies have since been conducted to understand how clays participate in chemical evolution processes [[3], [4], [5], [6]]. This is due to their advantages, which include the following: (a) their ordered arrangement [7,8], (b) their large adsorption capacity [3,9,10] (c) their protective role regarding energy sources (*e.g.,* gamma radiation), [4], (d) their ability to concentrate organic chemicals [8], and (e) their ability to serve as templates during polymerization [[11], [12], [13], [14]]. In addition, clays are widely distributed, they are historically prevalent during the entire timeline of geological and biological events on Earth, and they have a strong affinity for organic molecules such as nitrogenous bases [3,15,16].

**Fig. 1:**
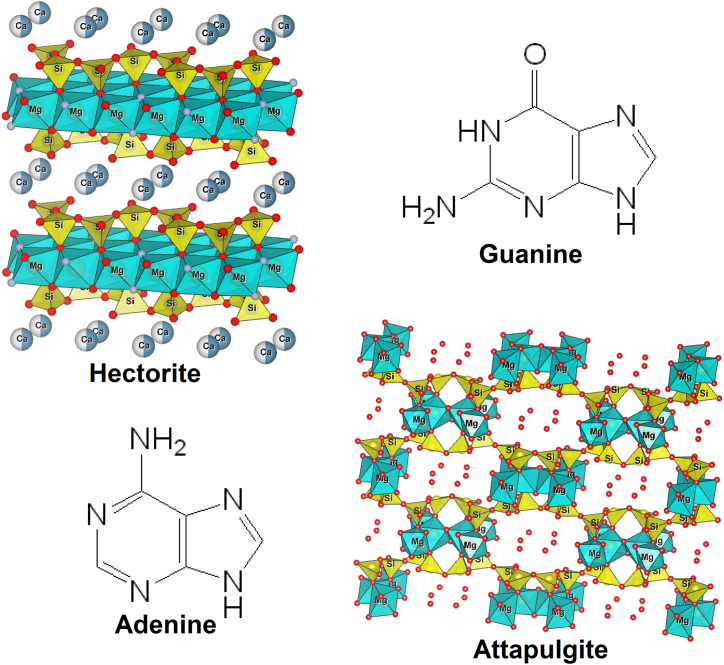
Molecular structure of hectorite (top-left), guanine (top-right), adenine (bottom-left) and attapulgite (bottom right).

The most studied clays in chemical evolution processes are phyllosilicates. Smectites are a subgroup of phyllosilicates that includes montmorillonite, a clay widely used in origin-of-life studies [17]. Smectite clays are characterized by a 2:1 layer structure with an expandable interlamellar space, which depends on the number of unoccupied octahedral sites. Therefore, smectites are classified into two groups: dioctahedral and trioctahedral. Hectorite (Fig. 1), a product of hydrothermal reactions [18], is a predominantly trioctahedral clay [19]. The octahedral site usually has divalent cations (*e.g*., Ca^2+,^ Fe^2+^, Mg^2+^). Si^4+^ and Al^3+^ are mainly present in the tetrahedral site [3]. Hectorite has an ideal molecular formula of Na_0.3_Mg_2.7_Li_0.3_Si_4_O_10_(OH)_2_ and a cation-exchange capacity (CEC) of 120 m_eq_ 100 g^−1^ [20], which is a measure of the total negative charges within the clay that could adsorb organic cations such as nitrogenous bases.

In contrast, attapulgite (Fig. 1), also known as palygorskite, has different geological origins: (a) sedimentary, (b) found in hydrothermally altered volcanic rocks, and (c) originates from a smectite precursor (*e.g.,* montmorillonite) [21,22]. Thus, we included this clay in our experiments on prebiotic chemistry processes. Attapulgite, with the ideal formula of (Mg_2_Al_2_)Si_8_O_20_(OH)_2_·4H_2_O, is in the sepiolite-palygorskite group (2:1 layer composition commonly with lath or fibrous morphology) [23], and its structure resembles a channeled wall with every second brick missing [24]. It is a dioctahedral clay formed in elongated, discontinuous, octahedral layers that alternate with continuous tetrahedral layers. The octahedral layers consist of magnesium partially substituted with aluminum or iron sandwiched between the (SiO_4_) tetrahedral layer and Al(OH)_3_ octahedral unit [25]. The CEC of attapulgite is 30–40 m_eq_ 100 g^−1^ [26], and it is one half to one third of that of montmorillonite [24].

Adenine and guanine (Fig. 1) are purine bases in deoxyribonucleic acid (DNA) and ribonucleic acid (RNA). Due to their participation in many bioorganic compounds, their role in chemical prebiotic processes has been widely studied. Some have proposed that both nitrogenous bases may have been present in terrestrial and extraterrestrial primitive environments [[27], [28], [29], [30]]. The purine-based nucleosides (adenosine and guanosine) show a much higher radiolysis resistance than the pyrimidine-based nucleosides (cytidine and uridine) when the irradiation is in a solid state and in vacuo at room temperature to a total dose of 3.2 MGy [31]. Adenine has been synthesized in abiogenic experiments from the polymerization of concentrated ammonium cyanide solutions [27,[32], [33], [34]]. Other prebiotic syntheses of adenine have been proposed, including formation from formamide under catalytic conditions [3,35]. The polymerization of concentrated ammonium cyanide solutions has also produced guanine but it at yields 10 to 40 times less than that of adenine. Its prebiotic synthesis would apply to frozen regions of the primitive Earth or other extraterrestrial bodies [36]. Adenine, hypoxanthine, and guanine can be produced from heated (130 °C), UV-irradiated formamide solutions without an inorganic catalyst, as Barks et al., 2010 [37] demonstrated.

Terrestrial or extraterrestrial primitive environments have been exposed to ionizing radiation from several sources as ^4^ K, ^232^Th, ^235^U, ^238^U, ^244^Po [[38], [39], [40]]. Therefore, the compounds in those environments may also have been exposed to these energy sources. To understand the effects of this, the radiolysis of bioorganic compounds has been studied from the chemical evolution perspective. Adenine and guanine radiolysis has been widely studied [17,[41], [42], [43], [44], [45]]. However, the nitrogenous bases' absorption mechanism has been studied for different minerals and under different physicochemical conditions [3,9,15,46]. Only a few experiments have evaluated clays’ protective character against high-energy radiation for nitrogenous bases adsorbed on the mineral surface [17,[46], [47], [48], [49]].

Experiments on organic matter absorbing into clays at different pH values are essential to understand the interaction mechanism between the solid mineral and the organic matter (*i.e.* physisorption and chemisorption). In addition, varying the pH values in these experiments simulates the different environments that probably existed on primitive Earth, for example, shallow water lagoons, submarine and subaerial hydrothermal systems, lakes and oceans [27]. Low pH values in the experiment could represent the Archean ocean with values between 4.8 and 6.5, a hypothesis based on that the early ocean was in equilibrium with an atmosphere composed mainly of CO_2_ [50]. The ocean's pH could have been as low as 3.35 near black smokers, as Schultz et al., 1997 [51] proposed. Currently, the effluent of black smokers is typically acidic (pH 2–3); however, the off-axis vents of black smokers are radically different and are highly alkaline (pH 9–11) [52]. On the early Earth, chemical weathering of land masses composed of mafic and ultramafic rocks would have been abundant; therefore, they would have released alkali cations and formed alkaline waters [53].

Studying organic compounds in the presence of clay minerals and under exposure to ionizing radiation fields is necessary for extending the knowledge of the possible roles that minerals may play as protectors of organic matter in terrestrial an extraterrestrial primitive environment, *e.g.,* submarine and subaerial hydrothermal systems. In this work, we studied the protection character of two clays in systems exposed to gamma radiation, simulating the early environments’ high radiation field. We selected adenine and guanine because they have been detected in terrestrial and extraterrestrial primitive environments [30,54,55] We selected hectorite and attapulgite due to their wide distribution and age. They are abundant in surface liquid water, during hydrothermal activity, and in chemical environments ranging from alkaline to neutral. Finally, to simulate microenvironments with high radiation activity in the early days of Earth (*e.g.,* high energy resulting from radioisotopes of ^4^ K, ^235^U, ^238^U, and ^232^Th), gamma rays from ^60^Co were used to simulate the early environment [40,49,56]. Gamma rays from ^60^Co are a convenient source of ionizing radiation and would be an excellent candidate for replicating GeV proton irradiation [57]. The effect of beta or alpha radiation in an organic compound mainly produces the same products, only in different yields. The high penetration of gamma rays allowed, the irradiations are in the bulk of solutions, and the radiation chemical processing is easier in gamma sources than other radiation sources.

## 2 Experimental

### 2.1 Material and method

The adenine (C_5_H_5_N_5_), guanine (C_5_H_5_N_5_O), sodium hydroxide (NaOH), potassium hydroxide (KOH), formic acid (CH_2_O_2_), ammonium hydroxide (NH_4_OH), nitric acid (HNO_3_), sulfuric acid (H_2_SO_4_), and hydrochloric acid (HCl_aq_) used in this work were from Sigma Aldrich® and have the highest degree of commercial purity available. The clays were obtained from the University of Missouri–Columbia, Source Clay Minerals Repository. Attapulgite (PFI-1) was collected from Gadsden County, Florida, USA, and hectorite (SHCa-1) from San Bernardino County, California, USA. The clays were characterized by attenuated total reflection Fourier transform infrared spectroscopy (ATR-FTIR) and X-ray diffraction (XRD). No pretreat was made to clays before the adsorption-desorption of organic matter processes. The water used to make all the solutions was deionized in a Milli-Q® Plus Millipore ultrapure water system.

### 2.2 Solution samples

Seven adenine solutions were prepared at different molar concentrations: 8 × 10^−6^, 9 × 10^−6^, 1 × 10^−5^, 2 × 10^−5^, 5 × 10^−5^, 9 × 10^−5^, and 1 × 10^−4^ mol L^−1^. Each solution's absorbance was measured with a UV–vis spectrophotometer at 260 nm. Seven solutions at different molar concentrations of guanine were also prepared: 2.5 × 10^−5^, 3 × 10^−5^, 4 × 10^−5^, 4.5 × 10^−5^, 5.5 × 10^−5^, 6 × 10^−5^, and 1 × 10^−4^ mol L^−1^. The solutions' absorbance was measured at 273 nm. The molar extinction coefficient was determined using the Lambert–Beer equation. Measurements were made in triplicate.

### 2.3 Analysis

#### 2.3.1 UV–vis spectroscopy

Adenine and guanine solutions standards and the supernatant of the adsorption–desorption experiments were analyzed using UV–vis spectrophotometry. The analyses were performed in a Varian® Cary 100 Scan spectrophotometer using 0.5 and 1 cm quartz cells.

#### 2.3.2 ATR-FTIR spectroscopy

To determine all changes in the solids, the samples were analyzed via FTIR spectroscopy in a Spectrum 100 (PerkinElmer) spectrophotometer with an ATR accessory. The samples were analyzed without prior treatment; 16 scans were made for each sample.

#### 2.3.3 HPLC-ESI-MS

Adenine and guanine solutions standards and the supernatant of the adsorption-desorption experiments were analyzed via liquid chromatography. The analyses were performed using an HPLC system (515 pump, Waters Corp.) along with a single quadrupole mass detection system (SQ-2 Waters Corp.) and an electronic distance measurement instrument (EDM) in positive mode (ESI^+^). It is sometimes necessary to ionize the samples to obtain a better response; therefore, the supernatants’ pH value was adjusted with formic acid or ammonium hydroxide. Working conditions were adjusted for a 1.58 kV capillary for adenine and 2.47 for guanine, a 19 V cone for adenine and a 39 V cone for guanine, at a temperature of 350 °C, and a desolvation gas flow of 650 L h^−1^ using a Symmetry C18 column (4.6 × 75 mm, size of 3.5 μm spherical particle, by Waters Corp.) under an isocratic elution with a mobile phase (80–20% methanol-water, HPLC-ESI-MS) and with a flow rate of 0.3 mL min^−1^. A fixed sample volume (20 μL) was injected using a loop. We performed HPLC-ESI-MS analyses on all the samples to ensure the presence of the adenine (136 *m*/*z*) and guanine (152 *m*/*z*) ions after each adsorption–desorption process. The retention time for adenine was 2.09 min, and for guanine, it was 1.57 min.

#### 2.3.4 X-ray diffraction (XRD)

Powder X-ray diffraction spectra were acquired with a Bruker D8 Advanced diffractometer equipped with a Ni filter and CuKα radiation at 40 kV and 40 mA. The measurement was made in the 2θ angular range from 3° to 90° with a 0.002° step scan and integration of at 2θ angles from 15 to 2° by 52 min.

### 2.4 Adsorption experiments

Different samples were prepared according to the following procedure. First, 3 mL of adenine (1 × 10^−4^ mol L^−1^) or guanine (1 × 10^−4^ mol L^−1^) solutions were mixed with 0.1 g of clay (hectorite or attapulgite), and then the mixture was shaken at 300 rpm for 5, 10, 15, 30, 45, 60, and 120 min. To evaluate the effect of pH and time on the adsorption processes, other experiments were conducted with adenine and guanine (1 × 10^−4^ mol L^−1^) at pH 1.5 (obtained by adding HCl_aq_) and pH 11 (obtained by adding a sodium hydroxide solution). After shaking, all samples were centrifuged at 20,000 rpm for 90 min. The pH was measured for all samples. The percentage of adsorption in all cases was determined by comparing the supernatant with a standard solution. UV–vis and HPLC-ESI-MS were employed for the analysis.

### 2.5 Desorption experiments

Two solutions were tested for use in desorption experiments: potassium hydroxide (KOH; 0.1 mol L^−1^) and calcium chloride (CaCl_2_; 0.01 mol L^−1^). Four sets of samples were prepared for each solution (KOH or CaCl_2_). Solutions containing 3 mL of KOH or CaCl_2_ were mixed with 0.1 g of the clay containing nitrogenous bases. Then, the samples were shaken at 300 rpm for 30, 60, 90, and 120 min. After that, all samples were centrifuged at 20,000 rpm for 90 min. The recovery of adenine or guanine was determined after the KOH or CaCl_2_ treatment, and all the supernatants were evaluated with UV–vis spectroscopy and HPLC-ESI-MS analysis.

### 2.6 Gamma irradiation experiments

The glassware was treated with a warm mixture (1:1% v/v) of nitric and sulfuric acid for 30 min, followed by washing with distilled water and later heating in an oven at 300 °C overnight to minimize contamination [58]. Polyallomer Tic Wall brand centrifuge tubes (13.5 mL) were placed in a mixture of water and hydrochloric acid (1:1% v/v) for 24 h and completely dried.

In this work, two systems of nitrogenous bases adsorbed into clays were irradiated under different conditions: (a) adenine-clays systems in an aqueous suspension and (b) guanine-clays in the solid state. Solid state: the adsorption procedure under the conditions indicated in the previous section, the samples were centrifuged to separate the supernatant, while the clay was dried entirely at 50 °C.

**Fig. 2:**
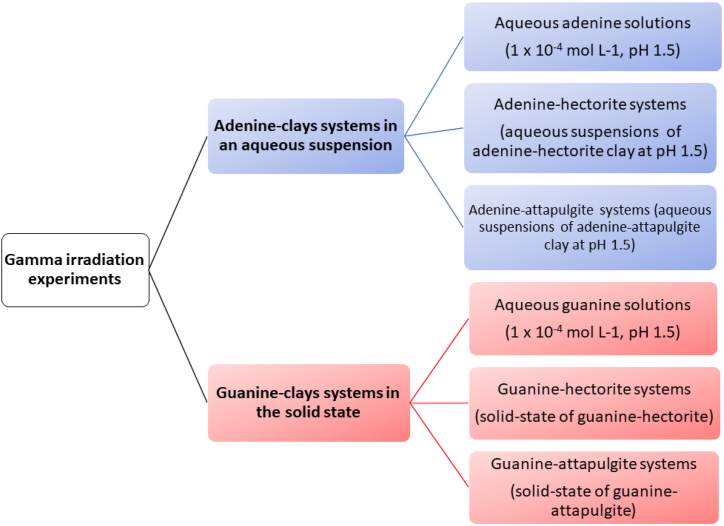
Adenine and guanine systems were exposed to gamma radiation fields under different conditions. Adenine and guanine systems are shown in blue and red, respectively.

To understand the radiolitic processes, different systems were studied for adenine and guanine (Fig. 2). All the systems were exposed to a gamma-ray source (Gammabeam 651 PT, Instituto de Ciencias Nucleares, UNAM) under an oxygen-free atmosphere at room temperature (298 K). The dose was evaluated using a ferrous sulphate-copper sulphate dosimeter [58]. The dose rate was 200 Gy min^−1^ at a fixed position at the gamma source. In this study we used nonconventional high doses for the systems, to simulate a high field radiation environment on primitive sceneries.

## 3 Results

### 3.1 Clays: hectorite and attapulgite characterization by XRD and ATR-FTIR

#### 3.1.1 XRD

The characteristic diffractograms for both clays were obtained. The XRD spectrum of hectorite showed the reflections at about 2θ = 7.38°, 20.87°, 29.42°, 35.99°, and 61.04°, which correspond to the (001), (110, 020), (004), (130, 200), and (060, 330) planes of hectorite and some small amounts of calcite impurities at about 2θ = 29.4° (104) [59,60]. The XRD spectrum of attapulgite showed reflections at about 2θ = 8.4, corresponding to a basal spacing between the (110) planes of 1.04 nm of the clay crystal lattice. The peaks in 13.84°, 16.39°, 19.84°, 20.86°, 26.63°, and 35.31° correspond to the primary diffraction of the Si–*O*–Si (200), (130), (040), (121), (400), (231), and (161) planes of attapulgite [61,62].

#### 3.1.2 ATR-FTIR spectroscopy

The FTIR spectra for both clays were obtained. Hectorite and attapulgite have several characteristic bands in the infrared region, which are listed in Table 1.

**Table 1:** Characteristic bands of clays in the infrared region.

Clay	Functional group	Wave number (cm^−1^)	Type of vibration
Attapulgite	(Mg/Al/Fe) O–H	3615	Stretching
Attapulgite	(Si)O–H	3548	Stretching
Attapulgite	Si–O–M	1699	Stretching
Attapulgite	Si–O–Si	1196	Stretching
Attapulgite	Si–O–Si	1030	Stretching
Attapulgite	Si–O–Mg	980	Stretching
Hectorite	Mg–O–H	3690	Stretching/overtone
Hectorite	(H)O–H	3442	Stretching
Hectorite	(H)O–H interlayer	1630	Stretching
Hectorite	CO_3_ (calcite)	1395	Stretching
Hectorite	Si–O	990	Stretching
Hectorite	CO_3_ (calcite)	872	Stretching

The presence of a calcite admixture in hectorite was confirmed in both the XRD and the ATR-FTIR data. The information provided by ATR-FTIR and XRD analyses was that along with the hectorite, there were also small amounts of calcite impurities.

### 3.2 UV–vis spectrophotometry calibration curves of nitrogenous bases

The maximum absorbances for adenine and guanine were at 260 and 273 nm, respectively. A linear relationship between concentration (mol L^−1^) and absorbance was determined for both systems. The molar extinction value determined for adenine was 14,770 cm^−1^ M^−1^, and for guanine, it was 8316.2 cm^−1^ M^−1^.

### 3.3 Effects of pH on the adsorption capacity

**Fig. 3:**
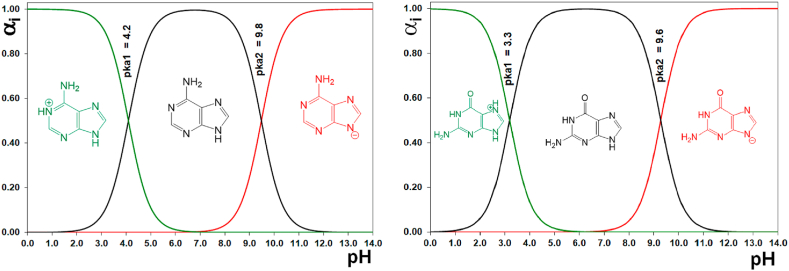
Species distribution diagram for adenine (left) and guanine (right) at different pH values.

A general method of modifying the clays is the traditional ion-exchange method. The inorganic cations are not strongly bound to the clay surface; therefore, organic cations replace the cations in the clay. Eventually, organic intercalants need only to be water-soluble, cationic, and stable under the reaction conditions [63]. For example, in the adsorption of adenine on sodium montmorillonite, the main mechanism of adsorption is the exchange of cations—among other interactions, such as van der Waals force—and hydrogen bonds [64]. Therefore, the site of nitrogenous base adsorption into clays can be explained by their predominant species at different pH values. At pH 1.5, the pKa_1_ of adenine and guanine are 4.2 and 3.3, respectively, and they are positively charged; at pH 11, the pKa_2_ of adenine and guanine are 9.8 and 9.6, respectively, and they are negatively charged, while both bases are neutral at pH 7 (Fig. 3).

The adsorption of the nitrogenous bases at different pH values into hectorite and attapulgite is attributed to pH and pKa, as will be discussed below.

**Fig. 4:**
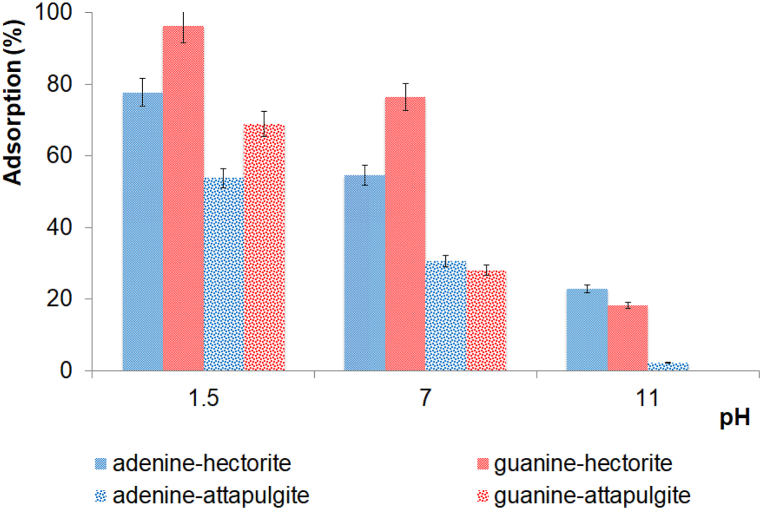
Percentage of adsorption of adenine and guanine into hectorite and attapulgite at different pH values (120 min of shaking).

Fig. 4 shows the results of the adsorption of adenine and guanine in hectorite and attapulgite at different pH values after 120 min of shaking. Adenine and guanine's absorption process into hectorite is rapid and likely starts when the nitrogenous base solutions come into contact with the clay. However, the first measure took place after 5 min of shaking to trigger the adsorption process, which depends on the pH value. For adenine into hectorite at pH 1.5, 7, and 11, the adsorption was 77.6%, 54.6%, and 22.8%, respectively. For guanine at pH 1.5, 7, and 11, the adsorption was 96.3%, 76.4%, and 18.3%, respectively. At pH 1.5, the species of nitrogenous bases are protonated according to their pKa_1_, and for this reason, the extent of the adsorption is the highest within the negatively charged interlamellar spacing. Hectorite is trioctahedral smectite, and the origin of the negative layer charge is less localized than it is in other smectites (*e.g*., dioctahedral smectites) such as montmorillonite. The less localized negative charge explains why there are different percentages of adsorption into the dioctahedral smectite and trioctahedral smectite. Perezgasga et al., 2005a [15] reported 95% adenine adsorption at acidic pH into Na-montmorillonite, and our results show only 77.6% adenine adsorption at acidic pH into hectorite. In addition, Paredes-Arriaga et al. (2021) [65] reported 100% guanine adsorption at acidic pH into Na-montmorillonite since the dominant specie is positively charged at acidic pH. There is an interchange of positive ions, and the main adsorption mechanism is ionic interchange. Our results showed 96.3% guanine adsorption into hectorite. Therefore, cation exchange successfully absorbed adenine and guanine due to Na^+^, Li^+^, and Mg^2+^, which are considered exchangeable cations for natural smectites.

For adenine and guanine into attapulgite, Fig. 4 shows the percentage of the adsorption of adenine and guanine into attapulgite at different pH value. From the results, the amount of adenine and guanine adsorbed into attapulgite decreased with an increasing pH solution due to the changes in the physicochemical properties of these molecules. The electrostatic point (IEP) is about 7, indicating that at a pH below 2, the nitrogenous bases carry positive charge, which leads to enhanced adsorption processes. However, at a pH above 11, nitrogenous bases were negatively charged due to the deprotonation of the nitrogen of the imidazole group. Increasing the pH may result in an increased electrostatic repulsive force, decreasing the adsorption of nitrogenous bases in clays.

The results show that the maximum percentage of adenine and guanine adsorption was at pH 1.5 in both clays. Table 2 shows the concentration of adenine adsorbed in both clays. These results could be derived from the clays’ structures (Fig. 1), and therefore, they could be derived from the high CEC value for hectorite and the large surface area of attapulgite.

**Table 2:** Concentration of adenine and guanine adsorbed into clays at pH 1.5.

	% adenine adsorbed at pH 1.5	Concentration (mol L^−1^)	% guanine adsorbed at pH 1.5	Concentration (mol L^−1^)
Hectorite	77.66	7.76 × 10^−5^	96.30	9.63 × 10^−5^
Attapulgite	54.89	5.48 × 10^−5^	68.86	6.88 × 10^−5^

*Initial concentration of adenine 1 × 10^−4^ mol L^−1^

The cationic species of nitrogenous bases at pH 1.5 interchanged with the guest ions in the clays' interlayer space. The neutral species of nitrogenous bases at pH 7 interacted with different sites of the clay surface, that is, in the interlayer space and at the crystal edges. Meanwhile, the anionic species of nitrogenous bases at pH 11 interacted at the clays’ edges positively charged. These different modes of interactions and their dependence on the pH conditions suggest the possibility of modulating not only the adsorption sites of the clay but also the desorption processes, which has important implications in the chemical evolution of nitrogenous bases in different primordial environments.

After the adsorption experiments, XRD and ATR-FTIR analyses were conducted to track the changes in our systems. The XRD analyses show that the clays did not suffer attacks by acidic and basic treatment. The bands present in the ATR-FTIR data corresponded to the N–C vibrations present in nitrogenous bases (Table 3).

**Table 3:** ATR-FTIR results of nitrogenous bases-clays systems.

Nitrogenous base-clay	pH adsorption	Functional group	Wave number (cm^−1^)	Type of vibration
Adenine-hectorite	7	N–H	3600	Stretching
Adenine-hectorite	7	N–C	1200	Stretching
Guanine-hectorite	1.5	N–H	3300	Stretching
Guanine-hectorite	1.5	N–C	1427	Stretching
Guanine-attapulgite	1.5	N–C	1450	Stretching

### 3.4 Desorption experiments

After the adsorption experiments at pH 1.5, we conducted experiments testing the desorption of nitrogenous bases from hectorite and attapulgite. The treatment consisted of shaking the nitrogenous base-clay systems with KOH or CaCl_2_ at different times. The presence of nitrogenous bases in the supernatant suggests an ion-exchange mechanism. UV–vis spectrophotometry and HPLC-ESI-MS analyses allowed us to confirm the nitrogenous bases’ chemistry identity after the desorption treatment.

#### 3.4.1 Hectorite

**Fig. 5:**
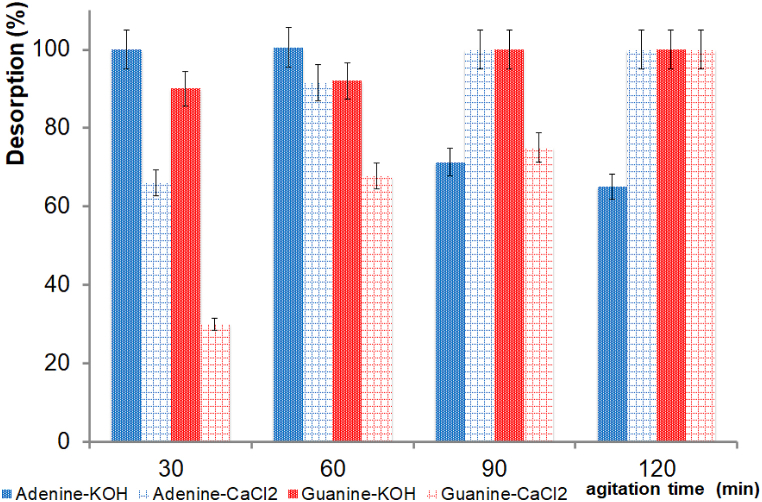
Desorption of adenine and guanine from hectorite, with KOH and CaCl_2_ as functions of shaking time.

We performed the recovery of the nitrogenous bases after a cycle of treatment with KOH or CaCl_2_ for the adenine-hectorite and guanine-hectorite systems. It was possible to de-adsorb all guanine and adenine from the clays after the shaking processes. The exchange of Ca^2+^, and K^+^ in hectorite indicates that an ion-exchange mechanism mainly governed the nitrogenous bases’ adsorption mechanism. The difference between KOH and CaCl_2_ in the desorption processes was the time to carry out the processes. For experiments performed with KOH, just 30 min of shaking were necessary to almost fully desorb adenine and guanine, while 100% of adenine and guanine were desorbed of hectorite with CaCl_2_ after 120 min of shaking (Fig. 5).

#### 3.4.2 Attapulgite

**Fig. 6:**
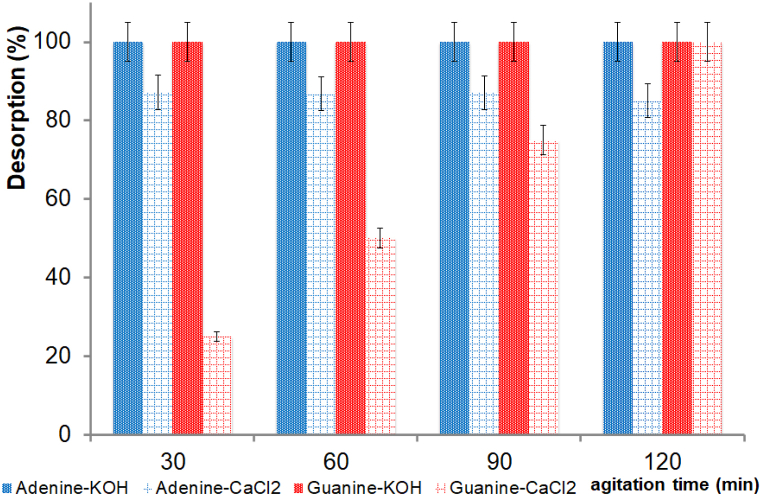
Desorption processes of adenine and guanine from attapulgite, with KOH and CaCl_2_ as functions of shaking time.

For the adenine-attapulgite and guanine-attapulgite systems, the adenine and guanine desorption processes were successful after being treated with KOH and CaCl_2_ for three cycles. For the KOH treatment, 100% of the adenine was desorbed, whereas for the CaCl_2_ treatment, 85% of the adenine was desorbed. For adenine, the shaking time of either treatment (KOH and CaCl_2_) did not affect the desorption processes (Fig. 6). When guanine was tested, 100% was desorbed with KOH after 30 min of shaking, whereas 70% was desorbed with CaCl_2_ after 120 min of shaking (Fig. 6). The exchange of Ca^2+^ and K^+^ in attapulgite indicates that the adsorption mechanism of nitrogenous bases is mainly governed by an ion-exchange mechanism.

Since the desorption processes with KOH were successful, all desorption experiments in this study were conducted with a KOH solution. However, nitrogenous bases can also be desorbed from clays with CaCl_2_.

### 3.5 Radiolysis experiments

It is important to study radiation-matter interaction in different media because any physicochemical variable can affect the reaction mechanisms in radiolytic processes. Therefore, in this work, we irradiated different systems of nitrogenous bases adsorbed into clays under various conditions. We used high doses of irradiation to simulate a high radiation field of ionizing radiation in primitive environments.

We determined the stability of the analyte (adenine and guanine) exposed to ionizing radiation under different conditions (aqueous suspension and solid-state) based on the percentage of the remainder after the radiolysis, which was conducted at different doses and at room temperature.

#### 3.5.1 Adenine aqueous solution

To understand the interaction of radiation and the nucleic acid bases under different conditions: we study different systems: adenine interacting with hectorite or attapulgite at pH 1.5 and a system without any clay (*i.e*., containing only adenine and water molecules).

After irradiating the adenine in aqueous solutions ranging from 1.5 to 36 kGy with gamma, the decomposition increased as the dose increased. At 36 kGy, less than 10% of the adenine was recoverable.

**Fig. 7:**
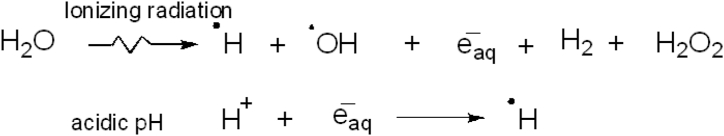
Water radiolysis of acidic systems.

Previous studies have explored in detail the radiation chemistry of adenine in aqueous solution [44,[66], [67], [68]]. Our results confirmed two effects: In diluted solutions, the main effect of radiation is a secondary effect from the water radiolysis products (Fig. 7) (1) At pH 1.5, which was used in the radiolysis process, the principal water radiolytic products without oxygen are •H and •OH. This last one is an oxidizing species [58,69] (Fig. 7).

**Fig. 8:**
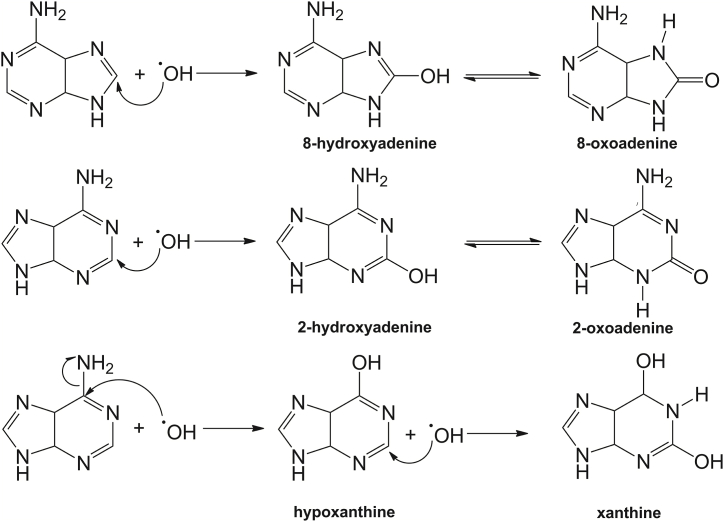
Proposed radiolysis mechanism of adenine in aqueous solution.

(2) The product of the interactions among this oxidizing species (mainly •OH with adenine) produces 8-oxoadenine, the tautomeric form of 8-hydroxyadenine, which is the main product of adenine radiolysis. However, from the HPLC-ESI-MS analyses, we have detected other radiolytic products, 2-oxoadenine (in low yields) and xanthine, as have previously been reported by other authors [41,43,44,47,70,71]. These radiolytic products were detected based on their ion mass and mass spectra fragmentation patterns. Fig. 8 shows the reaction mechanism, which demonstrates an inarguable advantage of ionizing radiation related to prebiotic chemistry due to the interconversion of nitrogenous bases [72]. However, this work did not identify and quantify the adenine radiolysis products.

#### 3.5.2 Adenine-clay systems

**Fig. 9:**
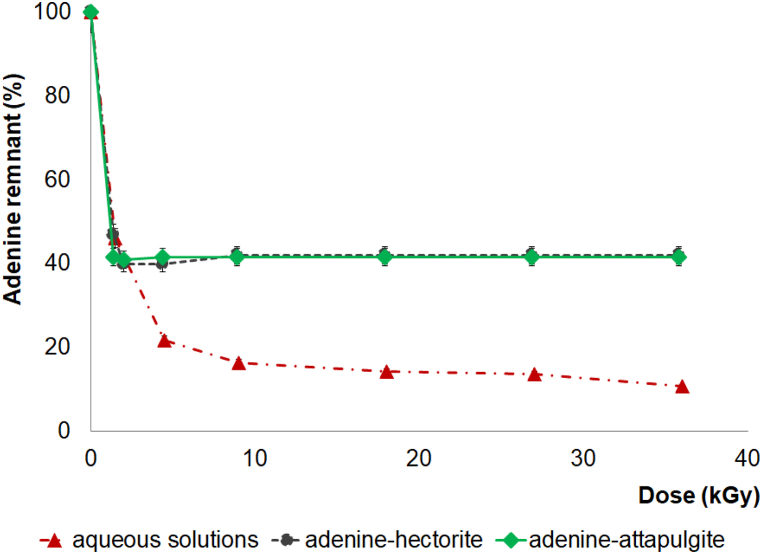
Recovery of adenine (%) as a function of the irradiation dose (kGy) in aqueous solutions and adenine-clay systems.

Following the gamma irradiation processes (ranging from 1.5 to 36 kGy) of the adenine–clay systems in aqueous suspension, three KOH desorption cycles were conducted to remove any remnant of adenine from the clay. The results show that 42% of the adenine was recovered from the adenine–clay systems, whereas only 10% of the adenine was recovered from the irradiated aqueous solution without clay. Therefore, the adenine decomposition rate in an aqueous solution without clay is higher than in an adenine–clay system (Fig. 9). However, both clays perform a protective role for adenine when the system is exposed to gamma radiation. In possible primordial conditions, the net effect will be an increase in adenine stability.

After the radiolysis and desorption processes, the remaining concentrations of adenine in the three systems should correspond to the percentage of adsorbed adenine at pH 1.5. Table 4 shows the percentage of adsorbed adenine and the final concentration of adenine recovered after gamma irradiation.

**Table 4:** Adenine remnant concentration after 36 kGy gamma irradiation-desorption processes.

	Initial adenine concentration (mol L^−1^)	Adenine concentration after radiation processes (mol L^−1^)
Aqueous solution	1 × 10^−4^	6.09 × 10^−5^
Hectorite	7.76 × 10^−5^	3.25 × 10^−5^
Attapulgite	5.48 × 10^−5^	2.26 × 10^−5^

#### 3.5.3 Aqueous guanine solutions

**Fig. 10:**
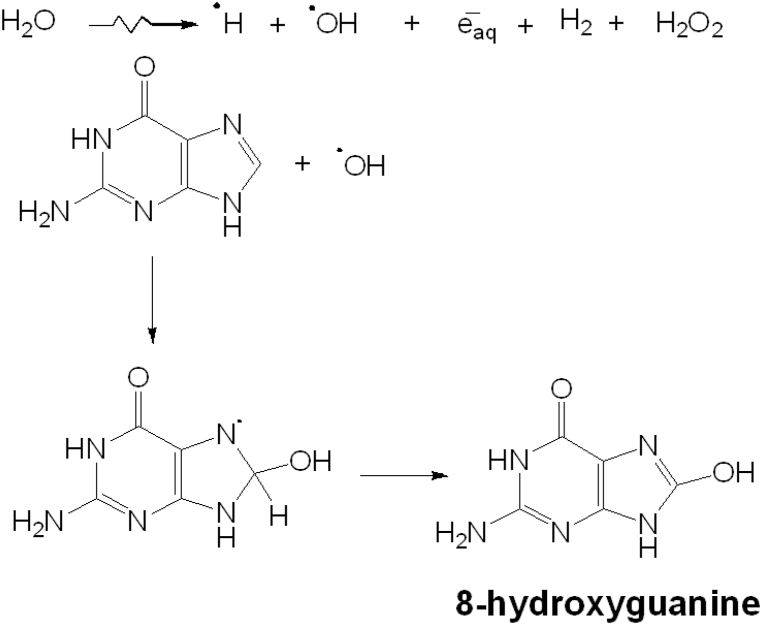
Proposed radiolysis mechanism of guanine in aqueous solution.

Radiolysis of guanine in aqueous solution (1 × 10^−4^ mol L^−1^) showed the same behavior as adenine and the results show that guanine is not very stable in an aqueous medium. We detected no remnant at a dose of 5 kGy since the molecule interacts with the species formed by the radiolysis of water. The main product of guanine radiolysis in an aqueous solution is 8-hydroxyguanine, which was determined by HPLC-ESI-MS. Fig. 10 shows the reaction mechanism. Water radiolysis (Fig. 7) produces the hydroxyl radical (•OH), which intervenes in the production of 8-hydroxyguanine.

#### 3.5.4 Solid state guanine

**Fig. 11:**
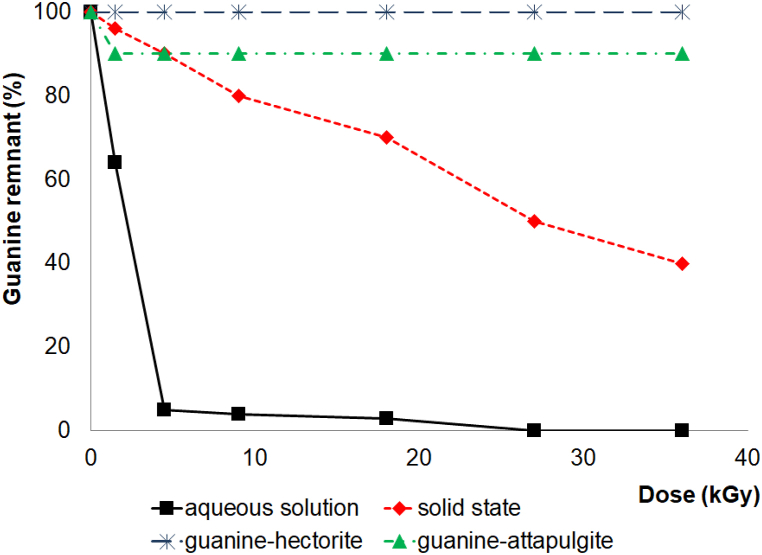
Recovery of guanine (%) as a function of the irradiation dose (kGy) in aqueous solutions, solid states, and guanine-clay systems.

When organic matter is irradiated in a solid state, the radiation generates radicals that stabilize in a solid matrix, allowing researchers to identify and quantify them. We irradiated solid-state guanine samples up to 36 kGy at a temperature of 298 K. After the gamma radiolysis, we made an aqueous solution of guanine (1 × 10^−4^ mol L^−1^). The aqueous solution samples were analyzed via UV–Vis and HPLC-ESI-MS, and a remnant concentration of 40% was observed after radiolysis (Fig. 11). The main reaction product was the dimer of guanine (*m*/*z* 301), which was obtained after 5 kGy of irradiation.

#### 3.5.5 Guanine-clay systems

After the adsorption of guanine–hectorite and guanine–attapulgite at pH 1.5, we conducted gamma radiolysis on solid-state samples to determine the effect of irradiation on the guanine–clay system. The results show that 100% of the guanine was recovered from the systems. The decomposition rate of the guanine in a solid state without clay is higher than in a guanine–clay system (Fig. 11). In both solid systems, clays were more protective of organic matter when the solid systems were exposed to gamma radiation. In possible primordial conditions, the net effect will be an increase in the nitrogenous bases’ stability.

After the radiolysis and desorption processes, the remaining concentrations of guanine in the four systems should correspond to the final concentration of guanine recovered after gamma irradiation (Table 5).

**Table 5:** Remaining guanine concentration after 36 kGy gamma irradiation-desorption processes.

	Initial guanine concentration (mol L^−1^)	Guanine concentration after radiation processes (mol L^−1^)
Aqueous solution	1 × 10^−4^	≈0
Guanine in solid state	1 × 10^−4^	4 × 10^−5^
Hectorite	9.63 × 10^−5^	6.19 × 10^−5^
Attapulgite	6.88 × 10^−5^	2.26 × 10^−5^

#### 3.5.6 Radiation chemical yield (G^0^)

The radiation chemical yield (G^0^) is the yield of decomposition or formation of a compound exposed to high-energy radiation. This value is determined by plotting the number of molecules formed or destroyed by 100 eV (G value) *vs*. the dose in eV mL^−1^. G^0^ is where the line intersects with the Y-axis. This value is independent of dose and concentration and, thus, facilitates consideration of the global damage caused by the radiation in the studied systems. A low value indicates that the systems are stable under the radiation and experimental conditions used, which is the case in this work. Although the nitrogenous bases rapidly decomposed in the solutions, the overall process was stable because their radiolytic products resulted in a reconstitution reaction.

Table 6 presents the G^0^ values observed for each system. These measurements were calculated using the extinction coefficient value of 14,386 cm^−1^mol L^−1^ for adenine and 8316.2 cm^−1^ M^−1^ for guanine. Fig. 9, Fig. 11 and the G^0^ values in Table 6 show that nitrogenous bases were more labile toward ionizing radiation when not adsorbed in clays. However, at doses of less than 5 kGy, all the systems had a similar decomposition slope, potentially due to the nitrogenous bases weakly bonded to the clays in the suspensions. After such low doses, each system stabilized, and the nitrogenous base concentration remained constant in the systems containing clay. In nitrogenous base solutions without clay, the decomposition was less pronounced. These results support the hypothesis that recombination reactions occur due to radiolytic nitrogenous base species [41,73]. However, the species remain close due to the clay structure, facilitating these recombination and regenerating the nitrogenous bases.

**Table 6:** G^0^ of the systems^a^^b^.

Sample	G^0^ value of 100 eV^−1^ molecules
Adenine aqueous solution	−0.60
Adenine-hectorite	−0.24
Adenine-attapulgite	−0.36
Guanine aqueous solution	−0.59
Guanine in solid state	−0.37
Guanine-attapulgite	−0.15
Guanine-hectorite	−0.13

G values are negative due that they are decomposition value.

a A yield-dose plot was used to derive an initial yield of decomposition [58].

b In SI-based units, the conversion factor is 1 molecule 100 eV^−1^ = 0.1036 μmol J^−1^.

## 4 Discussion

### 4.1 Nitrogenous bases adsorption experiments on clays

Hectorite is a smectite clay, which has physical and chemical properties relevant to the adsorption processes associated with prebiotic chemistry, such as the propensity to swell in water and other media (*e.g.,* artificial seawater), the capability to disperse in water, the capacity for cation exchange [[74], [75], [76]], and the ability to form organic and inorganic interlayer complexes. The inorganic cations on the hectorite surface (*e.g.*, Ca^2+^, Fe^2+^, Mg^2+^) could be replaced by organic cations through ion exchange, and the clay surface could become organophilic.

Since the structure of attapulgite differs from the structure of hectorite, we expected that the adsorption processes would also differ. Due to its three-dimensional structure, no montmorillonite-like swelling can occur [77]. However, the extremely large surface area of attapulgite (approximately 167 m^2^ g^−1^) makes it very absorptive in its natural form. The internal bundles, or haystacks, aid the external surface area in achieving great amounts of adsorption [77,78].

The adsorption process of adenine and guanine into clays is rapid and likely starts when the nitrogenous base solutions come into contact with the clay. The main mechanism is by ion-exchange. The maximum percentage of adenine and guanine adsorbed remained constant and did not change as shaking time increased up to a maximum of 120 min.

The adsorption phenomenon mechanism is known to function through exchangeable ions. Therefore, clays adsorb nitrogenous bases by exchanging their ions (*e.g*., Na^+^, Li^+^, Mg^2+^, Ca^2+^) with the protonated species of nitrogenous bases. Therefore, pH values do not impede nitrogenous base adsorption processes. In some alkaline environments, nitrogenous bases are adsorbed to a lesser degree, but the phenomenon persists. Some authors [[79], [80], [81]] have proposed that the evaporation of water as a consequence of carbonic and formic acid formation reactions could quickly modify the pH in microenvironments, generating a very acidic localized pH.

Among the favorable events in chemical evolution processes could be the increase in the concentration of nitrogenous bases and other compounds of biological importance, which, according to Miller (1953) estimates, reached concentrations of 10^−9^ mol L^−1^. Over time, and after the many changes that could have occurred in primitive Earth, mechanisms with participating clays were favored, as suggested by Bernal for the first time in 1949. Nitrogenous bases could have been adsorbed into the clay materials to increase their concentration. Since the nitrogenous bases would have been housed in the clays, they would have been protected from the radiation. Alternatively, this radiation could have triggered reactions with the clay acting as a catalyst. In other words, a series of favorable phenomena could have been initiated by adsorbing nitrogenous bases into the clays. In addition, adsorption would have protected a large portion of the nitrogenous bases present in the primordial soup from the extreme conditions of the primigenial Earth.

### 4.2 Nitrogenous bases desorption experiments

In this work, desorption experiments were conducted to evaluate clays’ ability to release nitrogenous bases under certain conditions. The processes were evaluated with KOH (0.1 mol L^−1^) and CaCl_2_ (0.01 mol L^−1^). The supernatants obtained from the desorption experiments were analyzed via UV–vis spectrophotometry and HPLC-ESI-MS to identify the desorbed molecules. According to the results, the KOH treatment was more effective than the CaCl_2_ treatment because the KOH solution desorbed 100% of the nitrogenous bases in only 30 min. This phenomenon can be explained by considering the pH value of the system: KOH 1 × 10^−4^ mol L^−1^ has a pH of 12, which favors the interchangeable ion (K^+^) process. To investigate the effect of ionic strength on desorption processes, CaCl_2_ was used without adjusting the pH. Fig. 5, Fig. 6 show that the desorption of nitrogenous bases increased by about 100% with increased shaking time (120 min) during the desorption processes with CaCl_2_. The clays tended to retain nitrogenous bases at low ionic strength conditions but tended to desorb them in acidic pH conditions. The lack of desorption under low ionic strength conditions can be attributed to the low competition for calcium due to its low concentration in system. A high calcium concentration could promote the desorption of nitrogenous bases from clays.

### 4.3 Gamma irradiation experiments on nitrogenous bases-clays

Similar to any physicochemical process, in all prebiotic processes, energy is fundamental because it promotes changes in matter. In general, energy plays an important role in chemical evolution since it is responsible for promoting and directing chemical reactions in any scenario. Among the main characteristics of ionizing radiation is its ability to deposit energy. When radiation interacts with matter, excited and ionized species are produced, which could generate specific chemical scenarios in prebiotic processes that are sometimes favorable, such as synthesizing organic matter and increasing the inventory of organic matter on early Earth, and are other times unfavorable, such as decreasing the stability of organic matter in early Earth scenarios. Therefore, the role of clays could be relevant and contribute to the stability of organic matter in primitive environments.

Clays may have played an important role as protective agents to prevent the organic molecules, like those studied in this research from degrading when exposed to high radiation fields. Fig. 11 shows that practically 100% of the guanine remained when it was adsorbed into the clays and exposed to an irradiation dose of 36 kGy. In the case of adenine, there was a remnant of 40% after it was adsorbed in the clays and irradiated at 36 kGy. In the absence of the clay minerals, guanine and adenine were decomposed under irradiation (Fig. 9, Fig. 11). The protective character of clays is a distinct advantage in primitive environments since the molecules produced by ultraviolet radiation, ionizing radiation, or electrical discharges have to be stable to interact with each other and form more complex molecules. Researchers are analyzing the protection mechanism and theorize that it may result from an energy transfer process [49]. Excitation, ionization, defects by radiation, storage, and energy-transfer processes inside the solid result from radiation's interaction with matter [82]. In multiphase systems, modes of energy transmission occur across the interface. Clays may store gamma radiation and transfer it at different wavelengths, decreasing breakdown [82]. However, energy transfer in heterogeneous catalysis is unclear. As a result, more study is required to appreciate its significance in prebiotic chemistry fully.

### 4.4 Relevance of clays’ organic interactions in chemical evolution processes

It is hard to imagine high concentrations of organic compounds were present during the early days of Earth; however, high concentrations are required for effective prebiotic synthesis. Consequently, experts have proposed that the adsorption process among organic matter and mineral surfaces could play a fundamental role as an organic concentrator. In our study, we successfully conducted adsorption experiments for both hectorite and attapulgite clays at different pHs. We not only discovered that adsorption processes are fundamental to prebiotic chemistry, but also concluded that desorption processes should be fundamental because they allow adenine and guanine to participate in other processes (*e.g.,* nucleotide production). In this work, the desorption of adenine and guanine with KOH or CaCl_2_ was successful. We used CaCl_2_ in the experiments due to the significance of salts in primitive environments, such as prebiotic seawater [48,83]. In addition, some Ca^2+^ ions could be important for forming peptides [84] and producing nucleosides [85]. The desorption of nitrogenous bases with a solution containing salts is advantageous because such processes are probably relevant in prebiotic environments that contain ions (*e.g.*, aerial hydrothermal systems or prebiotic seawater).

Radiolysis experiments showed that, in all cases, the presence of clay reduces the decomposition of nitrogenous bases more compared to radiation processes without clay. This effect could be related to energy transfer processes that contribute to the protective capability of clays [86]. Clays have been suggested as diffusion barriers to facilitate complex chemistry between the organic molecules adsorbed into them, including oligomerization reactions that are important from the perspective of molecular evolution [87].

Laboratory recreations of possible prebiotic systems and conditions are important to the study of the synthesis and stability of organic compounds. The role of clays in prebiotic processes has been considered since they act as simple adsorbents until the self-assembly processes of organic molecules take effect [3,13,15].

In this work, the principal approach is that clays could enable the concentration, storage, and release of organic compounds in prebiotic processes. Furthermore, they could protect organics when exposed to high radiation fields. The results showed that hectorite and attapulgite might have played an important role as protective agents. Clays avoid nitrogenous base degradation when they are exposed to high radiation fields. Therefore, nitrogenous bases could have undergone desorption and become available during the complex chemical evolution process.

## 5 Conclusion

Studying the synthesis and stability of organic matter exposed to ionizing radiation in different possible primitive environments is essential for understanding the chemical evolution and origin of life. We studied the adsorption-desorption processes of nitrogenous bases in hectorite and attapulgite clay minerals. The aim was to find the possible role of clays as protectors of organic matter when a system is exposed to high-energy radiation. The results highlight the protective character of clays toward organic matter exposed to ionizing radiation, as well as the accessibility of organic matter desorption processes in clay as a clear advantage in chemical evolution processes. According to these results, we propose that clays are crucial for the formation of increasingly complex molecules when preserving important molecules.

## Author contribution statement

Adriana Leticia Meléndez-López: Conceived and designed the experiments; Analyzed and interpreted the data; Wrote the paper.

Jorge Armando Cruz-Castañeda: Analyzed and interpreted the data; Wrote the paper.

Alicia Negrón-Mendoza: Conceived and designed the experiments; Analyzed and interpreted the data; Contributed reagents, materials, analysis tools or data; Wrote the paper.

Sergio Ramos-Bernal, Alejandro Heredia: Analyzed and interpreted the data; Contributed reagents, materials, analysis tools or data; Wrote the paper.

Laura Graciela Castro-Sanpedro, Dilan Victor Aguilar-Flores: Performed the experiments.

## Data availability statement

Data included in article/supplementary material/referenced in article.

## Declaration of competing interest

The authors declare that they have no known competing financial interests or personal relationships that could have appeared to influence the work reported in this paper.
